# Case Series: Can Daily Patient‐Reported Outcome Measures Be Implemented for Cancer‐Related Fatigue?

**DOI:** 10.1002/cnr2.70616

**Published:** 2026-06-23

**Authors:** Alexander Tilg, Andreas Meryk, Johannes G. Weiss, Roman Crazzolara

**Affiliations:** ^1^ Medical University of Innsbruck Innsbruck Austria

**Keywords:** cancer‐related fatigue, children, daily PROM, leukemia

## Abstract

**Background:**

Cancer‐related fatigue (CrF), a prevalent and debilitating symptom among cancer patients, remains underrecognized and unstandardized in routine oncology care. To address this, we introduced a new digital tool for daily CrF measurement and integration in routine cancer treatment.

**Case:**

This case series investigates the use of the ePROtect app for daily CrF assessment in two pediatric acute lymphoblastic leukemia (ALL) patients. Data were collected up to 2 weeks after the end of intensive therapy (before the start of maintenance) and analyzed using two questions informed by the National Comprehensive Cancer Network (NCCN), each offering five possible responses and categorizing fatigue into different subclasses. This analysis compares two 3‐year‐old girls with pre‐B ALL, one with standard risk (SR) and favorable genetics, the other one with poor therapy response and stratified to high risk (HR) treatment. The SR patient responded well to treatment, experiencing severe CrF on only 6% of the treatment days. In contrast, the HR patient required intensified treatment with multiple re‐induction phases, resulting in more frequent hospitalizations and severe CrF on 19.4% of the treatment days. CrF assessments were conducted daily for both patients, achieving 98.6% and 69.5% completion rates throughout intensive therapy, for the SR and HR patient, respectively.

**Conclusion:**

This case series illustrates the feasibility and potential suitability of our tool using PROMs for CrF assessment. Furthermore, it highlights the correlation between treatment intensity, complications, and CrF levels, underlying the importance of systematically measuring fatigue in cancer patients. Such measurement may inform future efforts to develop and refine individualized supportive care strategies.

## 1 Introduction

Cancer‐related fatigue (CrF) is one of the most prevalent and debilitating symptoms affecting both pediatric and adult cancer patients during and after treatment [1, 2]. Characterized by persistent physical, emotional, and cognitive exhaustion, CrF not only impairs daily functioning but also has long‐term consequences that may impact cancer survival [3]. Despite its clinical relevance, CrF remains underrecognized in both research and clinical practice, and its assessment is not yet standardized in routine oncology care. Eventually, this leaves patients vulnerable to the cumulative effects of untreated fatigue, which can significantly diminish their quality of life (QoL) [3, 4].

In order to understand the challenges involved, it is essential to examine how fatigue is measured. Fatigue detection varies widely, largely due to differences in the tools and methods [5]. Patient‐reported outcome measures (PROMs) are considered the gold standard for capturing the subjective nature of fatigue, but the appropriate language formulation and age‐specific adaptations have yet to be fully validated, particularly in children [4, 6]. Several challenges further complicate the integration of PROMs into daily practice: fatigue levels fluctuate over time, making it difficult to capture the full spectrum of the symptoms [1]. Furthermore, the burden of consistent PROM completion is overwhelming, particularly for patients with advanced disease or significant clinical deterioration [1, 7, 8].

To improve CrF management, we have introduced the app *ePROtect* for real‐time CrF monitoring in children undergoing cancer treatment [8]. This digital tool enables healthcare providers to identify dynamic changes in fatigue levels in real time and to consider timely supportive care actions in line with existing clinical guidelines and care pathways for CrF [9]. We illustrate how daily CrF measurement can be integrated into routine practice in these two cases, suggesting its potential role within a personalized care strategy.

## 2 Cases

### 2.1 Case 1—Standard Risk Leukemia

A 3‐year‐old girl was referred on February 2023, with a diagnosis of pre‐B ALL. Comprehensive molecular analysis identified favorable‐risk genetics, specifically the presence of *ETV6‐RUNX1*. The patient was enrolled in both the AIEOP‐BFM 2017 and ePROtect studies. Initial treatment involved a 4‐week induction phase consisting of glucocorticoids and including standard chemotherapy agents such as Vincristine, anthracyclines, Asparaginase, and intrathecal Methotrexate. The induction phase was well‐tolerated with no significant toxicities, allowing for the patient's discharge by week 3. However, at the end of week 4, she was readmitted for 5 days due to mucositis, requiring analgesic management.

Following completion of induction, a rapid clearance of leukemic blasts was observed, with minimal residual disease (MRD) testing negative by day 33. The patient then transitioned to outpatient care, where she received standard consolidation therapy until week 9. Two weeks later, an 8‐week phase of consolidation therapy began, alternating with high‐dose Methotrexate every 2 weeks. At week 25, re‐induction therapy was initiated, comprising a 3‐week course of glucocorticoids alongside Vincristine, anthracyclines, and Asparaginase, followed by a 2‐week post‐induction phase, including Cyclophosphamide, Cytarabine, Thioguanine, and intrathecal Methotrexate. The patient remained asymptomatic during re‐induction, only requiring a single 5‐day hospitalization phase at week 28 for management of neutropenic fever.

CrF assessments were completed by their parents on 98.6% of the 217 total treatment days, including 166 outpatient days (76.5%) and 51 inpatient days (14 days were unplanned admissions due to mucositis and febrile neutropenia). Mean completion time for the whole questionnaire including the CrF questions was 45.7 s (SD, 22.5). Severe CrF was reported on 2 weeks of therapy (6% of treatment duration) and correlated with the 3rd week of induction and the 4th week of re‐induction treatment. Moderate CrF was noted on 13.4% (preferably in temporal relation to the weeks before and after periods of severe CrF), mild CrF on 37.8%, and no CrF on 41.5% of the assessed treatment days (Figure 2 and Table 1).

**TABLE 1:** Overview of CrF rates compared among the SR and HR patients.

	Standard risk (days/percentage)	High risk (days/percentage)
Severe CrF (< 25%)	13 (6%)	84 (19.4%)
Moderate CrF (25%–49.9%)	29 (13.4%)	71 (16.4%)
Mild CrF (50%–74.9%)	82 (37.8%)	139 (32.1%)
No CrF (≥ 75%)	90 (41.5%)	7 (1.6%)
Missing data	3 (1.4%)	132 (30.5%)

### 2.2 Case 2—High Risk Leukemia

A 3‐year‐old girl was referred on August 2021, with a diagnosis of pre‐B ALL and negative cytogenetics. She was enrolled in the AEIOP‐BFM 2017 and ePROtect studies. The patient began immediately standard induction chemotherapy. However, by day 33, she demonstrated a poor response (no complete hematologic remission) to the induction regimen. Treatment was intensified with repeated re‐induction elements: HR1 at week 16, HR2 at week 19, HR3 at week 22 and 3× Protocol III at weeks 25, 41 and 53. Re‐induction phases incorporated glucocorticoids, Vincristine, Doxorubicin, Methotrexate and Asparaginase. At the end of each re‐induction phase, the patient experienced either mucositis or infectious complications, requiring immediate inpatient management. In total, the patient spent 284 (65.5%) days at home and 22 hospitalizations occurred (149 days), nearly half of them (48%) for unplanned admissions.

CrF assessment was completed on 69.5% of 433 total treatment days. CrF was not completed during periods of severe clinical deterioration, when the patient was unable to complete the forms together with their parents, or during phases of consistent well‐being at home. The mean time to complete the whole questionnaire was 60.8 s (SD, 27.0). Severe CrF was reported on 19.4% of the days, almost exclusively at prolonged induction phases containing glucocorticoids (Protocol I and three cycles of Protocol III). Moderate to mild CrF was observed in 48.5% of time and typically succeeded episodes of severe CrF or was reported after unplanned admissions for febrile neutropenia or mucositis. No CrF (1.6%) was documented only when the patient was at home and not receiving active treatment (Figure 2 and Table 1). Whereas phases of febrile neutropenia and mucositis were treated according to standardized protocols [10, 11], supportive interventions specifically targeting fatigue—including physiotherapy, occupational therapy, art therapy, and psychosocial support—were integrated into care and contributed to improvements in the child's overall physical and emotional well‐being.

### 2.3 Study Design

The CrF data collection of this case series was performed within a prospective, single‐arm longitudinal study which was started on May 1, 2020 at the Division of Childhood Oncology at the Medical University of Innsbruck and is still ongoing. Recruited parents for children under the age of five are instructed to complete daily PROMs online by using the ePROtect app. A description of ePROtect and its use in daily clinical practice has already been published [8, 12]. The Ethics Committee at the Medical University of Innsbruck approved this study (EC number: 1055/2020). Written informed consent is obtained from their parents.

### 2.4 Patient Selection

For the purpose of this study, we selected two patients with similar age (3 years of age) and disease (both with acute lymphoblastic leukemia), but different treatment intensity within the same clinical study protocol (EudraCT number AIEOP‐BFM ALL 2017: 2016‐001935‐12; one patient was treated for standard risk, while the other patient was treated for high risk leukemia). Data collection of CrF was started in both patients within the first week of treatment initiation and terminated 2 weeks after the end of intensive treatment (before maintenance). This limit was set due to high variability in completion rates after active treatment and to avoid falsification due to variable durations and low intensity during the maintenance period.

### 2.5 Description of CrF Assessment With ePROtect

**FIGURE 1:**
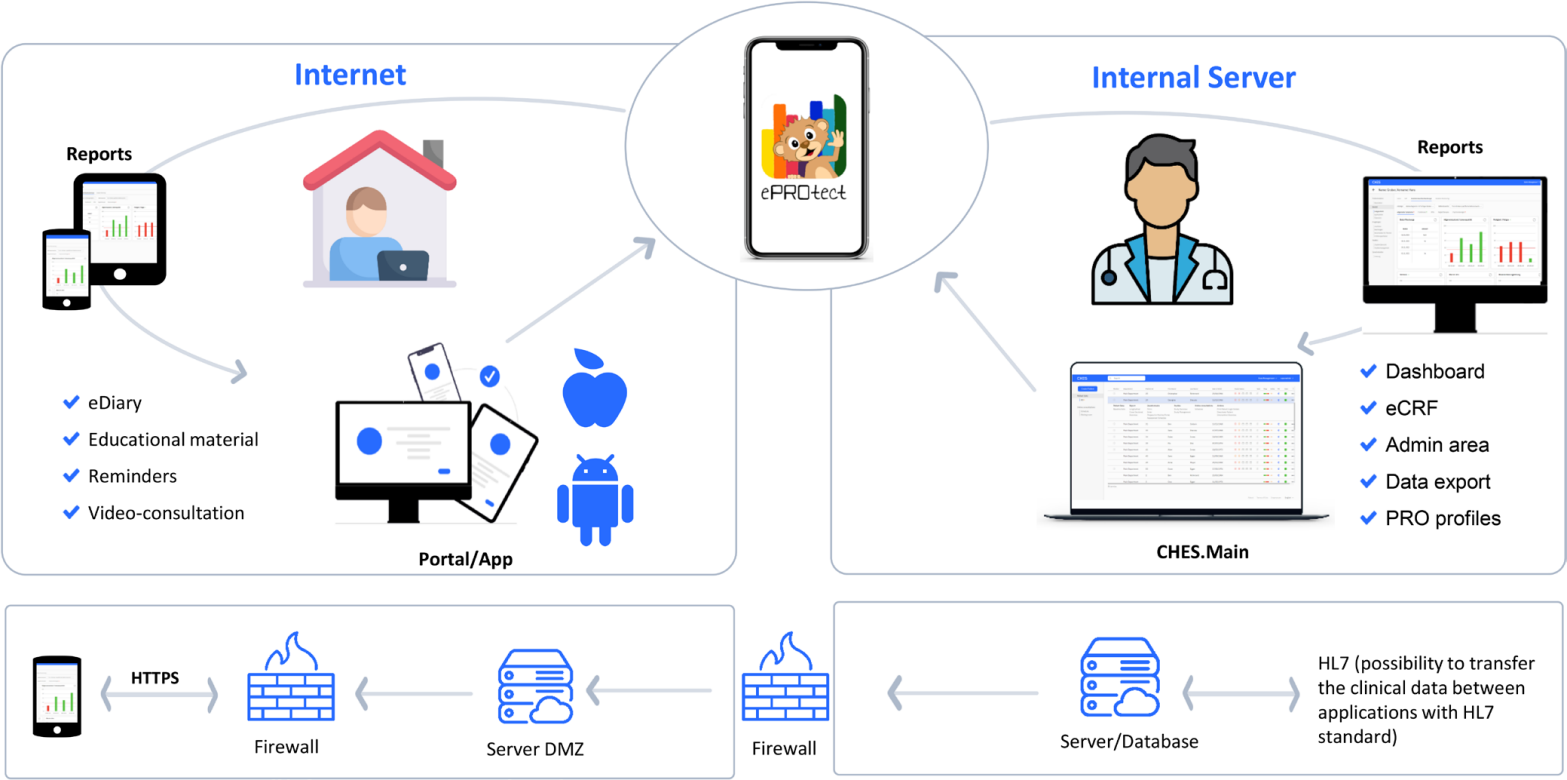
Daily CrF assessments and integration into clinical care. CrF is measured by 9:00 a.m. using ePROtect designed for real‐time monitoring. The collected data is processed through the computer‐based health evaluation system (CHES) on the internal server, enabling comparison with individual clinical information obtained from the clinical information system. Patients have access to their information system on firewall‐secured Internet, where an electronic diary and educational materials are provided.

Parents were offered participation in the ePROtect study, which included daily assessment of CrF via ePROtect. The assessment was conducted by 9:00 a.m. each day and was designed to capture real‐time data on CrF. The computer‐based health evaluation system (CHES) processed CrF measures and integrated it into a healthcare professional interface, allowing for comparison with individual clinical data obtained from the clinical information system (CIS) [13]. The CIS included comprehensive medical assessments, such as vital sign monitoring, physical examinations, and standardized laboratory tests (Figure 1).

As part of the daily PROM assessment, parents answered eight brief questions daily, focusing on CrF, pain, sleeping disorder, obstipation, nausea, and vomiting [10]. These questions were answered by parents using a five‐point scale for simplicity. Two of the eight daily questions directly targeted CrF: “Did your child feel too tired to play yesterday?” and “Was it difficult for your child to walk yesterday?” The items were conceptually aligned with the NCCN definition and screening recommendations for CrF [4]; however, CrF was assessed on a daily basis and exclusively via proxy report, we used a simplified 5‐point Likert scale to reduce response burden while maintaining comparability with older children, for whom the same scaling was applied. In addition, the application incorporates several features designed to enhance motivation and engagement, including gamification elements and reward mechanisms, age‐appropriate information about the disease, treatment and care setting, options for asking open questions, and the possibility of joining clinical visits via video, thereby supporting sustained participation of patients and families in daily monitoring. Our approach addressed both the degree of fatigue and its impact on daily activities.

### 2.6 Descriptive Analysis

**FIGURE 2:**
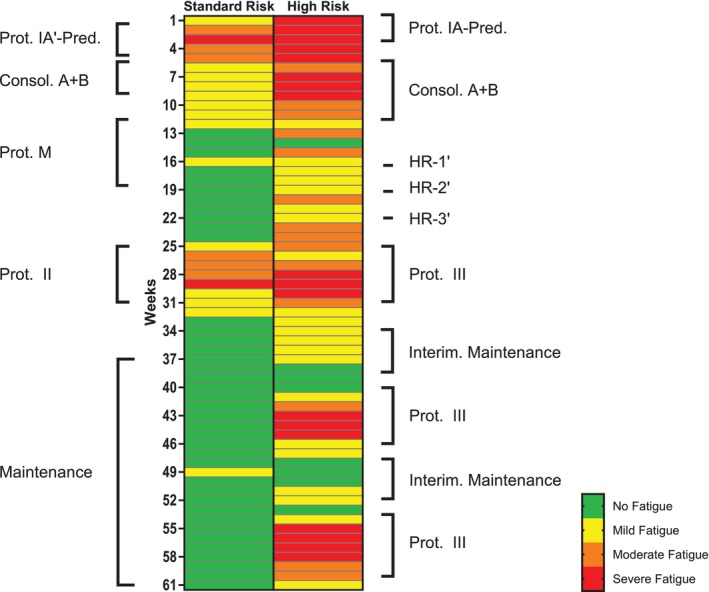
Weekly CrF levels in patients treated differently for ALL. The different CRF levels are visualized on a weekly basis and compared to different treatment phases (for standard‐risk [SR] and high‐risk [HR]), which are displayed on both *y*‐axes. For better visualization and comparability we included data from the maintenance therapy of the SR patient.

For each question, responses were given on a five‐point Likert scale. We calculated an average of the two CrF‐specific responses and categorized the results into four fatigue subclasses informed on the NCCN recommendations: 0%–25% impairment of physical activity as “no fatigue,” 25.1%–50% as “mild fatigue,” 50.1%–75% as “moderate fatigue” and 75.1%–100% as “severe fatigue.” Fatigue levels within 1 week were averaged and visualized in a heatmap using GraphPad Prism version 8.4 to display distribution (Figure 2).

## 3 Discussion

The implementation of daily CrF measurement in pediatric cancer patients appears to be feasible and valuable, as evidenced by the two cases reported in this study. Both patients were of similar age and shared the same underlying diagnosis (pre‐B ALL), but differed in their risk stratification and treatment intensity. Importantly, both demonstrated high completion rates of the PROMs, which strengthens the comparability and interpretability of the findings and represents a key feature of these two cases. Our data underscore the importance of this approach by several key areas. The daily CrF pattern closely correlates with known side effects of cancer treatment, particularly the subjectively very stressful therapy phase of glucocorticoid treatment and with phases of unplanned hospitalization for painful mucositis including potential opioids use and febrile neutropenia. In addition, reduced mobility and inability to walk may also be influenced by vincristine‐associated neurotoxicity and steroid‐induced myopathy, contributing to physical limitations that are not exclusively fatigue‐related [11, 14]. This highlights that the two‐question score captures overlapping aspects of CrF, including systemic fatigue and treatment‐related functional impairment.

Real‐time identification of these vulnerable phases may enable earlier intervention, anticipatory guidance, and effective treatment to reduce CrF symptoms. It may also enable physicians to differentiate which patients are at higher risk for severe CrF and related complications. This stratification is crucial for tailoring supportive care and allocating resources to those most in need.

The importance of obtaining CrF information through low‐barrier methods offers numerous advantages that enhance consistent reporting and improve overall care. The KLIK (Dutch: Kwaliteit van leven in Kaart) PROM portal and Symptom Screening in Pediatrics Tool (SSPedi) are other examples of how digital devices can be used for patients to increase their engagement in symptom reporting [15, 16]. However, these applications have been limited to longer reporting intervals (no prolonged daily approach) and have mostly been used for the purpose of study observations only [17, 18]. In our study, we have integrated CrF monitoring into clinical care by expanding interfaces and combining it with the existing clinical information system. The incorporation of gamification elements, age‐appropriate designs, disease, and treatment information empowers the older patients and families while streamlining data collection and analysis. The ability to visualize data enables real‐time monitoring and facilitates better communication between patients and healthcare providers. Moreover, these digital tools reduce the burden on families through remote accessibility and quick, convenient reporting. Compared to established fatigue‐specific instruments such as the Brief Fatigue Inventory and the Revised Piper Fatigue Scale [18, 19], ePROtect focuses not only on fatigue but enables integrated, longitudinal monitoring within routine care. In contrast to broader symptom assessment tools such as the Edmonton Symptom Assessment System Revised, EORTC QLQ‐C30, or PRO‐CTCAE, ePROtect is designed for real‐time, daily symptom monitoring integrated into clinical workflows [19, 20, 21]. This allows for continuous assessment of CrF alongside other relevant symptoms and may facilitate more timely, targeted interventions while enhancing patient engagement. In summary, ePROtect offers several strengths over traditional scales, including real‐time monitoring and enhanced patient engagement, allowing for a comprehensive assessment of fatigue's impact on daily life.

There are several limitations to this project and case series. Due to its implementation‐focused nature and the inclusion of only two patients, the findings may not be generalizable. In addition, the use of proxy‐reported PROMs represents a limitation compared to self‐reported measures, particularly because CrF affects cognitive, emotional, and physical domains that may not be fully captured by parent ratings. Nevertheless, for children under 5 years of age, proxy reporting remains the only feasible approach. Potential challenges related to the implementation of electronic CrF monitoring include data protection considerations and adherence to medical duty‐of‐care standards. Data protection issues can be mitigated through robust security measures, including code‐controlled access to the application, pseudonymization of patient data, and firewall‐protected data storage. Regarding medical duty of care, it is imperative to clearly define parameters to integrate symptom monitoring into routine clinical practice. Until the method is thoroughly validated, it should be considered a complementary tool rather than a replacement for standard care protocols. However, if symptom monitoring demonstrates significant clinical benefits, its omission from patient care could potentially be considered a deviation from best practices. Further research is needed to establish the validity and reliability of electronic symptom monitoring systems, determine optimal integration strategies within existing clinical workflows, assess the impact on patient outcomes and quality of care, and evaluate cost‐effectiveness and resource utilization. These investigations will inform evidence‐based guidelines for the responsible implementation of electronic symptom monitoring in pediatric cancer care.

In conclusion, CrF is a highly individual and complex symptom that occurs to a variable extent during cancer treatment. With the successful implementation of daily PROM assessments for CrF, we have a unique opportunity to display its variance and correlate it with short‐term factors like therapy phases, inflammation, or sleep. Further research in this area will contribute to a better understanding of CrF in cancer, ultimately leading to better symptom management and improved QoL.

## Author Contributions

**Alexander Tilg:** conceptualization, investigation, writing – original draft, methodology, validation, visualization, writing – review and editing, formal analysis. **Roman Crazzolara:** conceptualization, funding acquisition, writing – original draft, writing – review and editing, validation, methodology, project administration, supervision. **Johannes G. Weiss:** writing – review and editing. **Andreas Meryk:** conceptualization, investigation, writing – review and editing, writing – original draft, methodology.

## Funding

This work was supported by grants from the Kinderkrebshilfe Tirol und Vorarlberg and Kinderhilfe Südtirol‐Regenbogen.

## Conflicts of Interest

The authors declare no conflicts of interest.

## Data Availability

The data that support the findings of this study are available on request from the corresponding author. The data are not publicly available due to privacy or ethical restrictions.
